# 

**Published:** 1881-05-20

**Authors:** 


### TABLE OF CONTENTS.

ORIGINAL AND SELECTED ARTICLES.

Conversations upon the Physical and Mental Hygiene of Girlhood, by T. S. Powell, M.D, Atlanta, 161. Puerperal Convulsions, by T. S. Lallerstedt, M.D., of Georgia, 169. Rupture of the Urethra, etc., by Thos. R. Wright, M.D., of Georgia, 172. Syphilitic Phthisis in the Negro, by G. G. Roy, M D., Professor of Materia Medica in the Southern Medical College, 175.

ABSTRACTS AND GLEANINGS.

Treatment in cases of Excessive Lochial Discharges, 177. New Remedies, 178. Snake Bite, 181. Expert testimony in Toledo, 182. Parke, Davis & Co., 183, Ethylate of Sodium in Nævus, 184. Typhoid Fever; Coca in the Opium and Alcohol Habits, 185. Chlorate of Potassium: Anthracæmia, 186. Bi-Meconate of Morphia; Digitalis in Scarlet Fever, 187. Treatment of Itch; Asthma, 188. Atropia in Pregnancy; Diet in Corpulency; Atropia in Hysteria; Azotate of Ethyl, 189. Bromide Potassium; Quinia from Coal Tar; For Rheumatism; Membranous Croup, 190.

SCIENTIFIC ITEMS.

The Snake-Stones of Ceylon; Submarine Cables, 191. Cold Weather and Health; A New Test of Intelligence, 192.

PRACTICAL NOTES AND FORMULÆ.

Unknown Eruptive Diseases, 193. Diphtheria; Startin's Mixture; Gold Medal Cologne; Debell's Purgative Tincture, 194.

EDITORIAL AND MISCELLANEOUS.

Editorial Notices, 195. Medical Association of Georgia, 196. American Medical Association, 197. Book Notices, 199. Receipted; Special Notices, 200.

